# Sarilumab plus methotrexate improves patient-reported outcomes in patients with active rheumatoid arthritis and inadequate responses to methotrexate: results of a phase III trial

**DOI:** 10.1186/s13075-016-1096-9

**Published:** 2016-09-06

**Authors:** Vibeke Strand, Mark Kosinski, Chieh-I Chen, George Joseph, Regina Rendas-Baum, Neil M. H. Graham, Hubert van Hoogstraten, Martha Bayliss, Chunpeng Fan, Tom Huizinga, Mark C. Genovese

**Affiliations:** 1 Stanford University Medical Center, Palo Alto, CA USA; 2 Optum, Lincoln, RI USA; 3 Regeneron Pharmaceuticals, Inc, Tarrytown, NY USA; 4 Sanofi Genzyme, Bridgewater, NJ USA; 5 Leiden University Medical Centre, Leiden, The Netherlands; 6 Division of Immunology/Rheumatology, Stanford University School of Medicine, Palo Alto, California 94303 USA; 7 Mailing address: 306 Ramona Road, Portola Valley, California 94028 USA; 8 now with Novartis, East Hanover, NJ USA

**Keywords:** Rheumatoid arthritis, Sarilumab, Patient-reported outcomes, Interleukin-6, Fatigue

## Abstract

**Background:**

Sarilumab is a human monoclonal antibody directed against the alpha subunit of the interleukin-6 receptor complex. In the MOBILITY phase III randomized controlled trial (RCT), sarilumab + methotrexate (MTX) treatment resulted in clinical improvements at 24 weeks that were maintained at 52 weeks in adults with rheumatoid arthritis (RA), who have inadequate response to MTX (MTX-IR). These analyses indicate the effects of sarilumab + MTX versus placebo on patient-reported outcomes (PROs) in this RCT.

**Methods:**

Patients (n = 1197) were randomized to receive placebo, sarilumab 150 or 200 mg subcutaneously + MTX every 2 weeks for 52 weeks; after 16 weeks, patients without ≥20 % improvement from baseline in swollen or tender joint counts on two consecutive assessments were offered open-label treatment. PROs included patient global assessment of disease activity (PtGA), pain, health assessment questionnaire disability index (HAQ-DI), Short Form-36 Health Survey (SF-36), and functional assessment of chronic illness therapy-fatigue (FACIT-F). Changes from baseline at weeks 24 and 52 were analyzed using a mixed model for repeated measures. Post hoc analyses included percentages of patients reporting improvements equal to or greater than minimal clinically important differences (MCID) and normative values in the FACIT-F and SF-36. Pearson correlation between observed PRO scores and clinical measures of disease activity was tested at week 24.

**Results:**

Both doses of sarilumab + MTX vs placebo + MTX resulted in improvement from baseline by week 24 in PtGA, pain, HAQ-DI, SF-36 and FACIT-F scores (*p* < 0.0001) that was clinically meaningful, and persisted until week 52. In post hoc analyses, the percentages of patients with improvement equal to or greater than the MCID across all PROs were greater with sarilumab than placebo (*p* < 0.05), with differences ranging from 11.6 to 26.2 %, as were those reporting equal to or greater than normative scores.

**Conclusions:**

In this RCT in patients with MTX-IR RA, sarilumab + MTX resulted in sustained improvement in PROs that were clinically meaningful, greater than placebo + MTX, and complement the previously reported clinical efficacy and safety of sarilumab.

**Trial registration:**

ClinicalTrials.gov. NCT01061736. February 2, 2010

## Background

The initial focus of most randomized controlled trials (RCTs) of new therapeutic agents for rheumatoid arthritis (RA) is appropriately directed at reducing the symptoms and signs of disease, demonstrating reduction in the progression of structural damage, and improving physical function and health-related quality-of life (HRQOL). Crucial to the evaluation of a new therapeutic agent is the use of patient-reported outcomes (PROs) to comprehensively define treatment benefit as recommended by current international consensus [1–3].

This manuscript reports PRO data from the 52-week phase III MOBILITY RCT of sarilumab in combination with methotrexate (MTX) in patients with RA, who have inadequate response to MTX (MTX-IR) (clinicaltrials.gov identifier NCT01061736) [4]. Sarilumab is a human monoclonal antibody directed against the alpha subunit of the interleukin-6 (IL-6) receptor complex, which mediates pathways that contribute to joint inflammation and destruction, pain, and fatigue in RA [5, 6]. Clinical improvements including symptomatic, functional, and radiographic outcomes were observed at 24 weeks, as early as 2 weeks in some outcomes, and were maintained over the 52-week study duration; the most common treatment-emergent adverse events included infection, neutropenia, injection site reaction, and increased transaminase [4]. Current analyses evaluated the impact of sarilumab on PROs, and correlation between these and changes in disease activity.

## Methods

### Study design and population

The trial design and methods have been previously described [4]; in short, patients were randomized to receive subcutaneous placebo or sarilumab 150 mg or 200 mg every 2 weeks (q2w) in combination with MTX. Treatment duration was 52 weeks; on or after 16 weeks, patients without ≥20 % improvement from baseline in swollen or tender joint counts on two consecutive assessments or any other lack of efficacy based on investigator judgment were offered rescue therapy with open-label sarilumab 200 mg q2w. Efficacy was evaluated using three co-primary efficacy endpoints: American College of Rheumatology 20 % improvement (ACR20) response [1] at week 24, physical function at week 16 using the health assessment questionnaire disability index (HAQ-DI) [7], and change from baseline in radiographic progression [8] at week 52.

Inclusion criteria were age 18–75 years; fulfilment of ACR 1987 revised classification criteria for RA [9]; active RA (swollen joint count ≥6, tender joint count ≥8; high sensitivity C-reactive protein ≥0.6 mg/dl) despite stable dosing with MTX for ≥12 weeks; anti-citrullinated protein antibodies (ACPA) or rheumatoid factor (RF) positivity or presence of one or more documented bone erosions; or disease duration ≥3 months [4].

### Patient-reported outcomes

The patient global assessment of disease activity (PtGA), pain visual analog scale (VAS) and health assessment questionnaire disability index (HAQ-DI) were administered as part of the ACR response criteria [1] at baseline, weeks 2 and 4, and every 4 weeks thereafter. Functional assessment of chronic illness therapy-fatigue (FACIT-F) [10] was administered at baseline, weeks 2, 4, 12, 24, 36, and 52, and medical outcomes Short Form-36 (SF-36) Health Survey version 2 [11] was administered at baseline, and weeks 24 and 52 to evaluate general health status, also described as HRQOL. The FACIT-F includes 13 items rated by patients on a scale of 0–4 summarized as a total score of 0–52, with higher scores indicating less fatigue. The SF-36 evaluates eight domains (physical functioning (PF), role physical (RP, i.e., limitations due to physical health), body pain (BP), general health perceptions (GH), vitality (VT), social functioning (SF), role emotional (RF, i.e., role limitations due to emotional health), and mental health (MH)). For each domain, item scores are coded, summed, and transformed on to a scale from 0 (worst possible health state measured by the domain) to 100 (best possible health state). These domains are combined into physical component summary (PCS) and mental component summary (MCS) scores with normative means (SD) of 50 (10).

### Statistical analyses

The intention-to-treat (ITT) population was used in the current analyses. Changes from baseline at weeks 24 and 52 were analyzed using a mixed model for repeated measures (MMRM) that included treatment, prior biological use, region, visit, and treatment by visit interaction as fixed effects, and baseline score as a covariate; results are expressed as least squares mean (LSM) and standard error. In the MMRM analysis, for patients who required rescue, only data up to the time of rescue were included. Statistical significance was claimed only for those outcomes above the break in hierarchical testing used to control for multiple comparisons previously reported [4]. All other *p* values were tested without adjustment for multiplicity.

The proportion of patients reporting improvement from baseline at week 24 equal to or greater than the minimal clinically important difference (MCID) in HAQ-DI scores was determined using thresholds ≥0.22 [12] and ≥0.3 points, with both thresholds prespecified. Post hoc responder analyses were conducted to estimate percentages of patients who reported improvement from baseline equal to or greater than the MCID [12, 13] of 10 mm for PtGA and pain VAS scores [13–15]; 2.5 points for SF-36 PCS and MCS scores, 5 points for individual domains [16]; and 4 points for the FACIT-F [10]. In these responder analyses, patients who discontinued or received rescue medication were considered non-responders. The number-needed-to-treat (NNT) was calculated as the reciprocal of the difference in response rates between active treatment and placebo to obtain the outcome of interest in one patient, assessing the magnitude of the benefit obtained with treatment [17]. To further assess benefit, the proportion of patients who reported normative values in the SF-36 summary and domain scores and the FACIT-F were evaluated at week 24, as were those who reported values equal to or greater than the patient acceptable symptom state (PASS) thresholds in the six SF-36 domains for which it has been estimated (PF, 50; BP, 41; GH, 47; VT, 40; SF, 62.5; and MH, 72) [18]. The percentage of ACR20 responders who reported improvements equal to or greater than the MCID was determined post hoc. Correlation analysis (Pearson *r*) was performed to determine relationships between individual PROs and clinical measures of disease activity including 28-joint disease activity score using C-reactive protein (DAS28-CRP) and the clinical disease activity index (CDAI) at week 24. All analyses were performed using SAS version 9.2 (SAS Institute, Cary, SC, USA).

## Results

### Demographic and disease characteristics

Baseline characteristics were balanced across treatment groups (Table 1). Duration of RA ranged from 8.6 to 9.5 years and approximately 20 % of patients had previously received biologic disease-modifying anti-rheumatic drugs (DMARDs).

**Table 1:** Baseline demographic and clinical characteristics of the intention-to-treat population

Variable	Placebo + MTX (n = 398)	Sarilumab 150 mg q2w + MTX (n = 400)	Sarilumab 200 mg q2w + MTX (n = 399)
Age (years)	50.9 ± 11.2	50.1 ± 11.9	50.8 ± 11.8
Female (%)	80.7	79.8	84.5
Race (%)			
Caucasian	86.2	86.3	86.0
Black	2.5	2.5	2.0
Asian	8.0	8.3	8.3
Other	3.3	3.0	3.8
Region (%)			
Western Europe	18.6	18.8	18.8
South America	38.9	38.8	38.8
Rest of world	42.5	42.5	42.4
RA duration (years)	9.1 ± 8.1	9.5 ± 8.5	8.6 ± 7.0
Prior biologic DMARD use (%)	20.6	20.5	19.5
Seropositive for rheumatoid factor (%)	84.4	87.1	82.6
Anti-CCP antibody positive (%)	85.4	90.2	84.9
Tender joint count	26.8 ± 13.8	27.2 ± 14.2	26.5 ± 14.5
Swollen joint count	16.7 ± 9.3	16.6 ± 9.0	16.8 ± 9.7
CRP (mg/dl)	2.0 ± 2.3	2.4 ± 2.3	2.2 ± 2.4
DAS28-CRP	5.9 ± 0.9	6.0 ± 0.9	6.0 ± 0.9
PtGA (VAS)	63.7 ± 19.9	64.4 ± 20.4	66.3 ± 20.8
Pain VAS	63.7 ± 19.9	65.4 ± 21.4	66.7 ± 21.4
HAQ-DI	1.6 ± 0.7	1.6 ± 0.6	1.7 ± 0.6
FACIT-F	27.2 ± 10.4	26.3 ± 9.8	25.9 ± 10.4
SF-36 PCS	31.9 ± 6.9	31.5 ± 6.7	31.1 ± 6.8
SF-36 MCS	38.9 ± 11.4	39.0 ± 11.3	38.7 ± 12.0

Numbers are presented as mean ± SD unless mentioned otherwise. *q2w* every 2 weeks, *MTX* methotrexate, Anti*-CCP* anti-cyclic citrullinated peptide, *CRP* C-reactive protein, *DAS28-CRP* 28-joint disease activity score using C-reactive protein, *DMARD* disease-modifying anti-rheumatic drug, *FACIT-F* functional assessment of chronic illness therapy-fatigue scale, *HAQ-DI* health assessment questionnaire disability index, *SF-36* 36-item Short Form Health Survey-Version 2, *MCS* mental component summary, *PCS* physical component summary, *PtGA* patient global assessment of disease activity, *RA* rheumatoid arthritis, *VAS* visual analog scale

### Changes from baseline

**Fig. 1:**
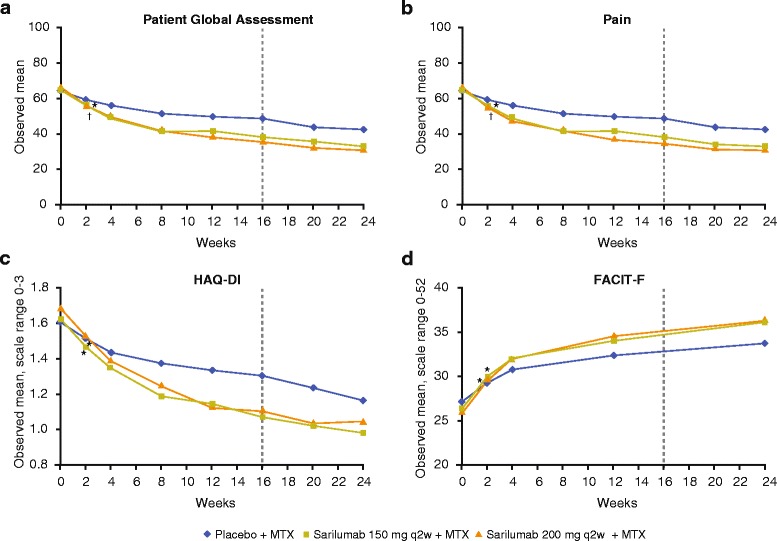
Mean scores at each visit through week 24 for **a** patient’s global assessment of disease activity, **b** pain, **c** physical function, and **d** fatigue. *Broken vertical line* indicates the earliest opportunity for rescue medication; patients who did not achieve ≥20 % improvement from baseline in swollen or tender joint count on two consecutive assessments were offered rescue therapy with open-label sarilumab 200 mg every 2 weeks. *HAQ-DI* health assessment, *FACIT-F* functional assessment of chronic illness therapy-fatigue questionnaire disability index, *MTX* methotrexate

LSM improvements from baseline at week 24 in the PtGA, pain, and HAQ-DI scores were greater with sarilumab 150 mg and 200 mg than placebo (*p* < 0.0001) and were maintained at week 52 (Table 2). The FACIT-F demonstrated improvement at week 24 with sarilumab 150 mg and 200 mg that was significantly greater than placebo and was maintained through week 52 (*p* < 0.0001 for both doses at both time points) (Table 2). Significant improvements were reported in the SF-36 PCS and MCS scores at week 24 with sarilumab compared with placebo (*p* < 0.05). Greater improvements were also observed with sarilumab in all eight domains at week 24 and at week 52 (*p* < 0.05) with the exception of the MCS and RE scores with sarilumab 150 mg at week 52 (Table 2). Improvements in PtGA, pain, HAQ-DI, and FACIT-F scores were evident by 2 weeks after the start of treatment (Fig. 1).

**Table 2:** Change from baseline in patient-reported outcome scores at weeks 24 and 52

Patient-reported outcome	(*n*) Least square mean ± standard error					
	Week 24			Week 52		
	Placebo + MTX (n = 398)	Sarilumab 150 mg q2w + MTX (n = 400)	Sarilumab 200 mg q2w + MTX (n = 399)	Placebo + MTX (n = 398)	Sarilumab 150 mg q2w + MTX (n = 400)	Sarilumab 200 mg q2w + MTX (n = 399)
PtGA	(253) -15.7 ± 1.4	(312) -28.3 ± 1.3***	(319) -32.9 ± 1.3***	(196) -20.3 ± 1.5	(272) -31.7 ± 1.4***	(272) -32.8 ± 1.4***
Pain VAS	(253) -15.4 ± 1.4	(313) -28.5 ± 1.4***	(321) -31.8 ± 1.3***	(196) -19.3 ± 1.6	(273) -32.7 ± 1.4***	(272) -33.1 ± 1.4***
HAQ-DI	(253) -0.32 ± 0.03	(313) -0.56 ± 0.03***	(316) -0.57 ± 0.03***	(195) -0.27 ± 0.04	(272) -0.62 ± 0.03***	(270) -0.63 ± 0.03***
FACIT-F	(252) 5.8 ± 0.5	(311) 8.6 ± 0.5***	(320) 9.2 ± 0.5***	(195) 6.1 ± 0.5	(270) 9.1 ± 0.5***	(271) 9.2 ± 0.5***
SF-36 component scores						
PCS	(246) 5.2 ± 0.5	(299) 8.0 ± 0.5***	(309) 8.4 ± 0.5***	(187) 5.6 ± 0.6	(257) 9.2 ± 0.5***	(263) 9.1 ± 0.5***
MCS	(246) 3.9 ± 0.6	(299) 5.7 ± 0.6*	(309) 8.2 ± 0.6***	(187) 5.5 ± 0.7	(257) 7.1 ± 0.6	(263) 8.4 ± 0.6**
SF-36 domain scores						
Physical functioning	(253) 11.9 ± 1.5	(312) 17.5 ± 1.3*	(316) 18.2 ± 1.3**	(195) 13.9 ± 1.6	(272) 21.3 ± 1.4**	(269) 21.3 ± 1.4**
Role physical	(252) 12.8 ± 1.4	(309) 18.7 ± 1.3**	(318) 20.4 ± 1.3***	(194) 15.5 ± 1.5	(266) 20.7 ± 1.3*	(271) 22.5 ± 1.3**
Body pain	(250) 15.3 ± 1.3	(312) 25.3 ± 1.2***	(318) 27.6 ± 1.2***	(192) 16.7 ± 1.5	(272) 28.1 ± 1.3***	(269) 28.0 ± 1.3***
General health	(248) 7.6 ± 1.1	(307) 12.80 ± 1.0**	(319) 15.2 ± 1.0***	(191) 10.5 ± 1.3	(269) 14.5 ± 1.1*	(271) 15.9 ± 1.1**
Vitality	(251) 9.8 ± 1.2	(308) 13.9 ± 1.1*	(320) 18.0 ± 1.0***	(194) 11.4 ± 1.3	(268) 17.5 ± 1.1**	(271) 17.7 ± 1.1**
Social functioning	(252) 9.8 ± 1.4	(312) 17.3 ± 1.2***	(320) 20.8 ± 1.2***	(195) 11.9 ± 1.6	(272) 20.4 ± 1.4***	(271) 20.8 ± 1.4***
Role emotional	(252) 10.3 ± 1.5	(308) 14.6 ± 1.4*	(318) 17.9 ± 1.4***	(193) 14.8 ± 1.6	(264) 17.3 ± 1.4	(269) 21.4 ± 1.4*
Mental health	(251) 7.4 ± 1.1	(308) 10.4 ± 1.0*	(320) 14.0 ± 1.0***	(194) 9.8 ± 1.2	(268) 13.0 ± 1.1*	(271) 14.3 ± 1.1*

*q2w* every 2 weeks, *FACIT-*F functional assessment of chronic illness therapy-fatigue scale, *HAQ-DI* health assessment questionnaire disability index, *SF-36* 36-item Short Form Health Survey-Version 2, *MCS* mental component summary, *MTX* methotrexate, *PCS* physical component summary, *PtGA* patient global assessment of disease activity, *VAS* visual analog scale. *p < 0.05, **p < 0.001, and ***p < 0.0001 versus placebo + MTX

**Fig. 2:**
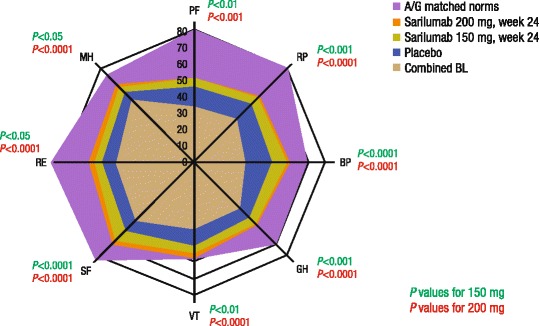
Combined baseline (*BL*) and post-treatment scores at week 24 across all Short Form 36 (SF-36) domains relative to age-adjusted and gender-adjusted norms (*A/G* matched norms) for the US general population. All scores on a 0–100 scale (0 = worst, 100 = best). *PF* physical functioning, *RP* role physical, *BP* body pain, *GH* general health, *VT* vitality, *SF* social functioning, *RE* role emotional, *MH* mental health. Note, as combined baseline scores are presented, change from baseline for each cohort cannot be inferred from Fig. 2 alone

As shown in Fig. 2, the SF-36 mean baseline domain scores were approximately 20 to 50 points lower than an age-matched and gender-matched normative US population, as a benchmark comparison, indicating substantial impairment of general health status. At week 24, patients receiving both sarilumab doses reported greater improvement from baseline versus placebo across all eight domains (*p* < 0.05), and VT scores approached normative values.

### Responder analyses

**Fig. 3:**
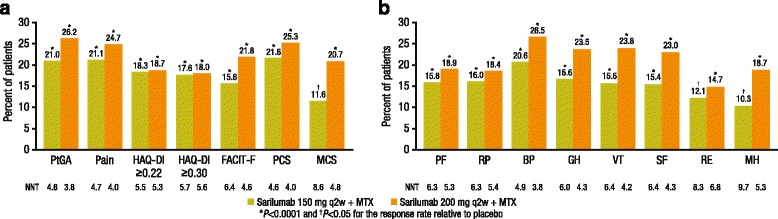
Responder analyses for patients with improvement equal to or greater than the minimal clinically important difference (MCID). **a** Differences from placebo in the percentage of patients reporting improvement equal to or greater than the MCID after 24 weeks of treatment according to patient global assessment (*PtGA*), pain, functional assessment of chronic illness therapy-fatigue (*FACIT-F*), health assessment questionnaire-disability index (*HAQ-DI*), and the Short Form 36 (*SF-36*) physical and mental component scores. **b** Differences from placebo in the percentage of patients reporting improvements equal to or greater than the MCID after 24 weeks of treatment in SF-36 domain scores. *PF* physical functioning, *RP* role physical, *BP* body pain, *GH* general health, *VT* vitality, *SF* social functioning, *RE* role emotional, *MH* mental health, *NNT* number needed to treat for sarilumab + methotrexate (*MTX*) versus placebo + MTX

In post hoc analyses, the percentages of patients reporting improvement equal to or greater than the MCID were higher with both doses of sarilumab than placebo across all PROs (*p* < 0.05), resulting in a NNT ranging from 4.0 (PCS for sarilumab 200 mg) to 8.6 (MCS for sarilumab 150 mg) (Fig. 3a). The percentage of patients who reported improvement equal to or greater than the MCID in individual SF-36 domains was consistently higher with both doses of sarilumab versus placebo for all domains (*p* < 0.05) (Fig. 3b); the NNT ranged from 3.8 (BP with the sarilumab 200 mg dose) to 9.7 (MH with the sarilumab 150 mg dose). The majority (59.4–89.8 %) of ACR20 responders reported clinically meaningful improvement across PROs.

**Fig. 4:**
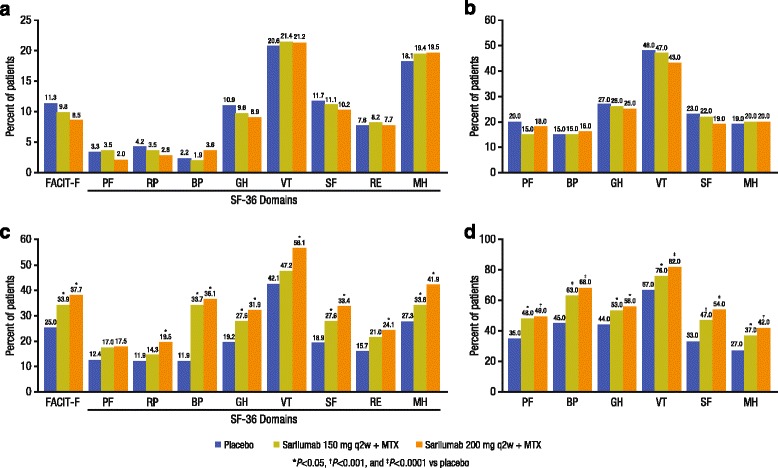
Responder analyses for normative scores and patient acceptable symptom state (PASS). **a** Percentage of patients reporting scores equal to or greater than normative values on the functional assessment of chronic illness therapy-fatigue (*FACIT-F*) and Short Form 36 (*SF-36*) at baseline. **b** Percentage of patients reporting scores equal to or greater than PASS thresholds at baseline. **c** Percentage of patients reporting scores equal to or greater than normative values on the FACIT-F and SF-36 at week 24. **d** Percentage of patients reporting scores equal to or greater than PASS thresholds at week 24. *PF* physical functioning, *RP* role physical, *BP* body pain, *GH* general health, *VT* vitality, *SF* social functioning, *RE* role emotional, *MH* mental health

The percentage of patients reporting scores equal to or greater than normative values in the FACIT-F and SF-36 domains was low across treatment groups at baseline, ranging from 1.9 % for BP to 21.4 % for VT (Fig. 4a), although higher proportions reported values exceeding PASS thresholds (from 15 % for BP to 48 % for VT) (Fig. 4b). At week 24, the percentage of patients who reported scores equal to or greater than normative values across the FACIT-F and SF-36 domains was greater with sarilumab treatment in the individual domains of BP, GH, SF, and MH domains with 150 mg, and across all domains with 200 mg except PF (*p* < 0.05) (Fig. 4c). The percentage of patients reporting scores equal to or greater than PASS was also higher with both doses of sarilumab relative to placebo (*p* < 0.05) (Fig. 4d), and the percentage was higher than those who reported scores equal to or greater than normative values in each of these domains.

### Correlation analysis

**Fig. 5:**
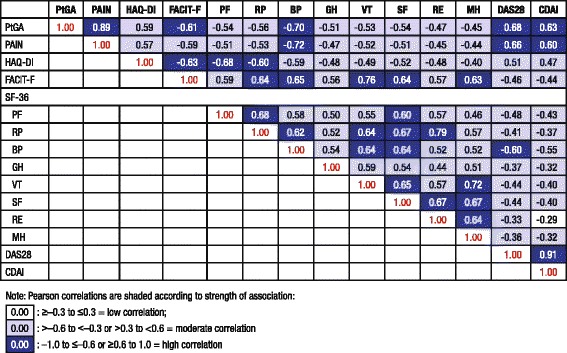
Correlation between observed patient-reported outcomes and disease activity scores at Week 24

At week 24, reported PRO scores demonstrated moderate to strong correlation with clinical measures of disease activity (DAS28 and CDAI) except for RE with the CDAI (Fig. 5). There was also moderate to strong correlation between PROs and individual SF-36 domains, with the strongest correlation between domains that measure similar constructs: the FACIT-F with VT (*r* = 0.76), HAQ-DI with PF (*r* = -0.63) and VAS pain with BP (*r* = -0.72).

## Discussion

In this phase III RCT, patients with moderate to severely active RA, who were MTX-IR reported that treatment with sarilumab + MTX resulted in improvements in pain, physical function, fatigue, and general health status that were clinically meaningful and greater than with placebo + MTX. These results complement the clinical efficacy previously reported [4].

There was concordance across PROs, with durable responses that appeared as early as 2 weeks in PtGA, pain, physical function, and fatigue scores, which were sustained through week 52. Improvements with 200 mg were generally greater than with the 150 mg dose. The FACIT-F scores showed significant and clinically meaningful improvement with sarilumab treatment; fatigue has a substantial impact in RA [19] and may be of greater patient concern than other signs and symptoms such as tender and swollen joints [20].

Responder analyses demonstrated benefit using a variety of approaches. In addition to reporting improvements equal to or greater than the MCID in PtGA, pain, HAQ-DI and FACIT-F scores that exceeded placebo, the proportions of responders at 24 weeks were greater across all PROs with both sarilumab doses than placebo. These responses resulted in a NNT ranging from 3.8 to 5.4 with sarilumab 200 mg, indicating that few patients would need to be treated to achieve clinically meaningful improvement. It is worth noting that the responder analysis conducted in this study was based on a conservative approach; patients who discontinued or received rescue medication were considered non-responders rather than as missing data.

As in other RCTs of biologic DMARDs [21–24], low baseline SF-36 scores indicated substantial impairment of general health status when compared with an age-adjusted and gender-adjusted US normative population, with significant improvements after treatment. Furthermore, using a higher level of response, i.e., improvement equal to or greater than the normative values for SF-36 PCS and MCS (≥50) and SF-36 domains based on this specific protocol population, were significant with sarilumab versus placebo. The achievement of normative values is also a more meaningful response than PASS, which represents a threshold of acceptability rather than demonstrating parity with an age-matched and gender-matched population, without arthritis or comorbidities. Together, these data indicate that active treatment with both doses of sarilumab improved health status and fatigue to levels commensurate with a patient population without arthritis or co-morbidities typical in RA.

Indeed, while correlation between symptoms/disease activity and functional outcomes suggested that clinical effects translate into patient-reported improvement in PtGA, pain, physical function and general health status, many of the correlations between the observed scores between PROs at week 24 were only moderate, indicating that these measures assess different domains of response and reflect relief from the broad burden of disease on patients’ lives.

A limitation of this study is that other than PtGA and HAQ-DI, all PROs were generic and do not specifically query about RA. However, all PROs utilized do assess concepts relevant to patients with RA and have been well-validated for use in RA. Additionally, the use of hierarchical testing procedures limited the ability to interpret some PRO data with regard to claims of statistical significance. Generalizability of the NNT estimates may also be limited because the comparator group, placebo + MTX, may not necessarily reflect clinical practice.

## Conclusions

In conclusion, reductions in disease activity with sarilumab treatment are associated with patient-reported benefits in global disease activity, pain, physical function, fatigue, and general health status. These effects, reported as early as week 2 and maintained over the 52-week trial duration, provide evidence of long-term benefits.

## Abbreviations

ACPA: anti-citrullinated protein antibody
ACR: American college of rheumatology
ACR20: American college of rheumatology 20 % improvement response
BP: body pain
CDAI: clinical disease activity index
DAS28-CRP: 28-joint disease activity score using C-reactive protein
DMARD: disease-modifying anti-rheumatic drug
FACIT-F: functional assessment of chronic illness therapy-fatigue
GH: general health
HAQ-DI: health assessment questionnaire disability index
HRQOL: health-related quality of life
IL-6: interleukin-6
LSM: least squares mean
MCID: minimal clinically important difference
MCS: mental component summary
MH: mental health
MMRM: mixed model for repeated measures
MTX: methotrexate
MTX-IR: methotrexate inadequate response
NNT: number needed to treat
PASS: patient acceptable symptom state
PCS: physical component summary
PF: physical function
PRO: patient-reported outcome
PtGA: patient global assessment of disease activity
q2w: every 2 weeks
RA: rheumatoid arthritis
RCT: randomized, controlled trial
RE: role emotional
RF: rheumatoid factor
RP: role physical
SD: standard deviation
SF: social functioning
SF-36: 36-item Short Form Health Survey
VAS: visual analog scale
VT: vitality
